# Transforming Care: Implications of Glucagon Like Peptide-1 Receptor Agonists on Physical Therapist Practice

**DOI:** 10.1093/ptj/pzaf061

**Published:** 2025-04-30

**Authors:** Julie Mulcahy, Anna DeLaRosby, Todd Norwood

**Affiliations:** Clinical MSK, Omada Health, San Francisco, CA, United States; Physera Physical Therapy Group, San Mateo, CA, United States; Clinical MSK, Omada Health, San Francisco, CA, United States

**Keywords:** Cardiovascular Diseases, Diabetes Mellitus, GLP-1, Medication Side Effects, Obesity, Pharmacology, Patient Care, Physical Therapists, Resistance Training, Weight Loss

## Abstract

The goal of this perspective is to bring awareness to the prevalence of glucagon-like peptide 1 receptor agonist (GLP-1 agonist) use, medication side effects, intervention considerations, and the role of the physical therapist in supporting their patients in their health journey when taking these medications. Management of obesity and diabetes is undergoing significant change with the increasing prevalence of GLP-1 agonist medications. This class of medications, which 1-in-8 adults in the United States report having taken, is becoming a critical component of obesity management, affecting the physiology and psychology of weight loss in novel ways. Due to the prevalence of musculoskeletal conditions in patients with diabetes and obesity, physical therapists can play a crucial role in the comprehensive care of patients on GLP-1 agonist therapy. This perspective explores the practice implications for managing patients living with obesity and/or diabetes who are taking GLP-1 agonists by describing the impact of GLP-1 agonists; the challenges of GLP-1 agonist use; and the considerations for recommending physical activity to patients using these medications. Physical therapists are well equipped to assist this population of patients by implementing strategies that enhance mobility, alleviate pain, prevent injury, mitigate lean muscle mass loss, and promote metabolic health, while adapting to the evolution of health and function patients experience while on GLP-1 agonist medications. As use of these drugs is expected to expand to other health conditions, there is a pressing need for physical therapists to adapt their practices to support the long-term health goals of their patients and ensure optimal patient outcomes.

## BACKGROUND

An unprecedented shift is occurring in the treatment of obesity and diabetes due to a new class of medications, glucagon-like peptide 1 (GLP-1) agonists, often referred to as GLP-1 agonists. As of March 2020, 41.9% of the United States population was classified as obese. Obesity is a risk factor for poor overall health, reduced functional capacity, decreased quality of life and is associated with a myriad of chronic conditions.^1^ Currently, more than 140 million Americans meet the prescribing criteria for GLP-1 agonist medications and 1-in-8 adults in the United States say they have taken a GLP-1 agonist medication, making it inevitable that all health care providers, including physical therapists, will treat patients taking these medications.^2^ To put the rapid increase in utilization of these medications in perspective, GLP-1 agonists are forecasted to have sales of approximately $50 billion in 2024, the largest annual sales figure ever for a class of drugs, and expected to surpass $100 billion in annual sales by 2029.^3^ Additionally, this class of drugs is being explored for the treatment of other diseases, demonstrating the need for all health care professionals to adjust care strategies to the needs of patients utilizing GLP-1 agonist medications. The most well-known medication names that fall into this class of drugs include:

- Dulaglutide (Trulicity)
- Liraglutide (Victoza)
- Liraglutide (Saxenda)
- Semaglutide (Ozempic)
- Semaglutide (Rybelsus)
- Semaglutide (Wegovy)
- Tirzepatide (Mounjaro)
- Tirzepatide (Zepbound)

Given the rapid increase in utilization of GLP-1 agonists and the substantial number of individuals who fit the clinical criteria to benefit from these medications, it is paramount that physical therapists are well informed about GLP-1 agonist medications. This paper explores the role of the physical therapist as part of an integrated approach to GLP-1 agonist management for diabetes and obesity. While these medications are being explored in the treatment of other conditions, the effects of these medications are most well understood in patients with obesity and/or diabetes. The evidence regarding those populations is the focus of this paper.

## MUSCULOSKELETAL EFFECTS OF DIABETES AND OBESITY

Patients with obesity and/or diabetes have an increased prevalence of musculoskeletal conditions. Approximately 90% of individuals with obesity have a musculoskeletal condition and 58% of those with diabetes have a musculoskeletal condition.^4^^,^^5^ As GLP-1 agonist medications are recommended to be used in conjunction with diet and exercise, addressing musculoskeletal concerns is essential for optimizing physical function and promoting sustainable health outcomes amidst the broader benefits and challenges associated with GLP-1 agonist use.

Diabetes and obesity, while primary cardiometabolic diseases, have significant negative effects on muscles, tendons and joints. Systemic inflammation associated with obesity and diabetes leads to inefficient muscle function, sarcopenia,^6^^,^^7^ and fatty or fibrous infiltrations of the muscle^7^ which reduce strength and contractile function of muscles.^8^ Individuals with type 2 diabetes mellitus are 4 times more likely than peers to experience tendinopathy.^9^ Tendons may be rigid with poorly organized collagen and decreased elasticity, which compromises their ability to tolerate load.^10^^,^^11^ Chronic inflammation can accelerate the degeneration of weight bearing joints and development of osteoarthritis (OA).^12^ Additionally, metabolic abnormalities are strongly correlated with OA in weight-bearing and non-weight-bearing joints, suggesting that the pathogenesis of OA is linked to oxidative stress and inflammation, which are hallmarks of cardiometabolic disease.^13^ While GLP-1 agonist-induced weight loss can decrease mechanical load through the musculoskeletal system and improve aspects of physical function, the resulting negative effects on tendons, impaired muscle function, and joint degradation can persist after a healthy weight has been achieved. Such impairments of the musculoskeletal system can prevent sustainable lifestyle changes, including regular physical activity, which make it difficult to achieve long term health maintenance post-GLP-1 agonist use.

Outside of its role in movement, skeletal muscle also plays an important role in glucose regulation. Lean muscle has more efficient glucose uptake, is more sensitive to insulin changes, and has better oxidative capacity than muscles that have fatty infiltration.^14–16^ Myokines secreted by skeletal muscle are involved in inter-organ crosstalk to promote insulin secretion, regulate mitochondrial function, increase lipolysis, and promote glucose oxidation to create energy.^16^ However, the onset of obesity results in lipid accumulation within muscles, impairing glucose regulation mechanisms.^16^ Obesity also promotes persistent inflammation, which causes fibrotic tissue to infiltrate muscles^7^ and surrounding connective tissue, impairing tissue structure thus decreasing the release of myokines and the cascade of positive effects they have on glucose regulation.

## MECHANISM OF ACTION

**Figure 1:**
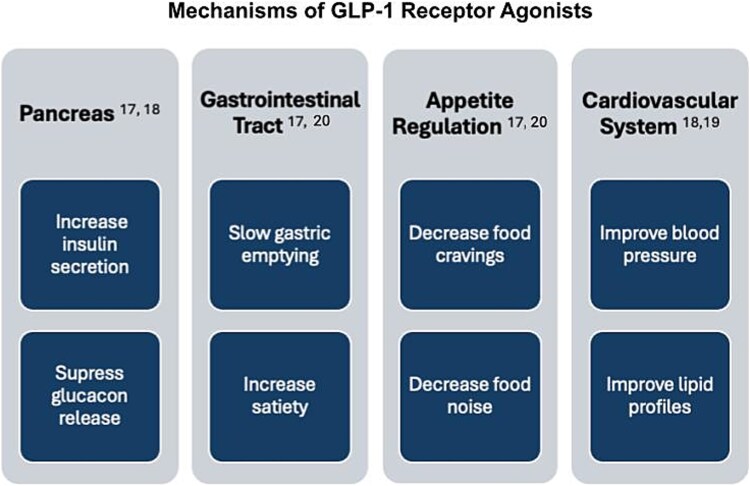
Mechanisms of GLP-1 Receptor Agonists. GLP-1 receptor agonists have many physiological mechanisms which impact multiple body systems. This figure represents the most well documented effects, but there are likely many other effects that will be more understood with continued research. Abbreviation: GLP-1 = glucagon-like peptide 1 receptor.

Glucagon-like peptide 1 receptor agonists mimic the action of naturally occurring GLP-1 agonist, a hormone secreted by the lower gastrointestinal (GI) tract in response to ingestion of food.^17^ When used to treat obesity and diabetes, GLP-1 agonists enhance insulin secretion from the pancreas to regulate blood sugar levels. Additionally, they suppress the release of glucagon, further improving glycemic control.^17^^,^^18^ Other actions within the GI system lead to slowed gastric emptying, which improves a sense of satiety and leads to decreased appetite.^19^^,^^20^ Decreased hunger leads to decreased calorie intake and weight loss. Outside of the GI system, GLP-1 agonist medication actions lower blood pressure and improve lipid profiles, though the mechanisms of these actions are not fully understood.^19^ Additional medication effects have been documented on other body systems such as the reproductive system,^21^^,^^22^ renal system,^23^^,^^24^ and psychological and cognitive systems,^25^^,^^26^ immune system,^27^ however, not all mechanisms are well understood. See Figure 1: Mechanisms of GLP-1 Receptor Agonists.

## SYSTEMIC EFFECTS

**Figure 2:**
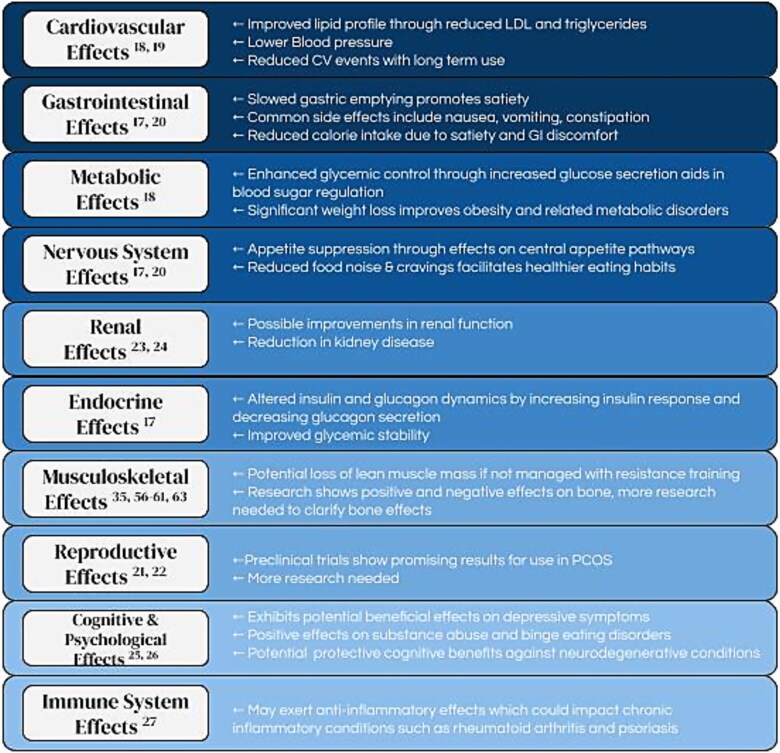
Systemic Effects of GLP-1 Agonist Medications. While GLP-1 agonist medications are now widely used for obesity and diabetes management, physical therapists will likely see increasing numbers of patients utilizing these medications to treat other conditions and should be familiar with their broad scope of action. This figure demonstrates the wide potential impact of GLP-1 agonist medications across body systems. Many effects are still being researched and not all applications are fully understood at this time. Abbreviations: CV = cardiovascular; GI = gastrointestinal; GLP-1 = glucagon-like peptide 1 receptor; LDL = low-density lipoprotein; PCOS = polycystic ovarian syndrome.

Research regarding the application of these medications is ongoing. See Figure 2: Systemic Effects of GLP-1 agonist Medications.

### Benefits of GLP-1 Receptor Agonists

**Figure 3:**
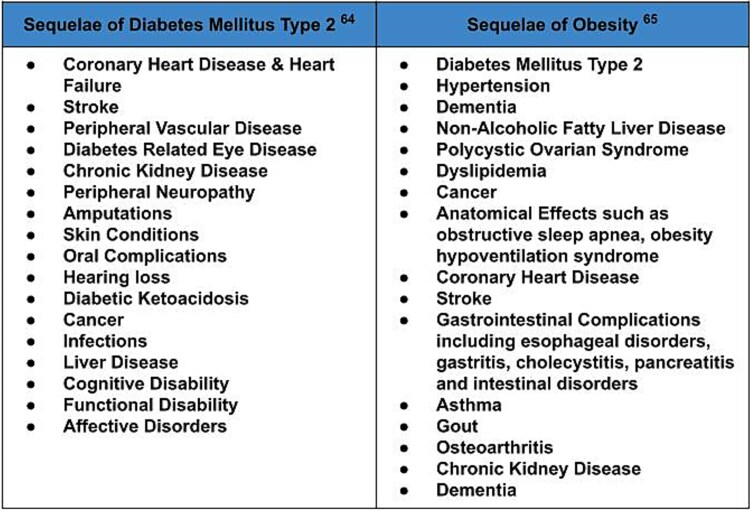
Sequelae of Diabetes Mellitus Type 2 and Obesity. This figure provides a broad overview of the potential health effects of living with diabetes mellitus and obesity. GLP-1 agonist medications effectively treat diabetes and obesity and stop the progression of disease leading to secondary health impacts. Abbreviation: GLP-1 = glucagon-like peptide 1 receptor.

GLP-1 agonists are a powerful tool in the management of diabetes and obesity. These medications lead to an average weight loss of 15% to 20% body weight and contribute to health improvements such as lower blood pressure, improved lipid profiles, and reduced risk of cardiovascular events.^17–19^^,^^28–31^ GLP-1 agonists have a profound effect on overall health trajectory by reducing the risk of serious health conditions and their numerous sequelae. Figure 3: Sequelae of Diabetes Mellitus Type 2 and Obesity provides an overview of the conditions associated with diabetes and obesity. By treating the primary diagnosis, the progress of downstream health impacts is significantly reduced.

Prior to the introduction of these medications, long term maintenance of weight loss was difficult due to the challenges of sustaining behavior change.^32^ Patient adherence to calorie restriction often wanes over time and many patients experience cycles of weight loss and gain.^20^^,^^33^ This cycle is disrupted for those who take GLP-1 agonist medications, as these medications help diminish “food noise,” allowing patients to adopt and maintain healthier eating habits.^20^ “Food noise” refers to the constant stream of thoughts, impulses, and distractions related to food. It encompasses mental clutter associated with food cravings, desires, decisions about what to eat, when to eat, and how much to eat. By reducing biological aspects of appetite and cravings through GLP-1 agonist medication, positive behavioral changes are significantly more manageable.

GLP-1 agonists significantly advance the management of obesity and related health issues by offering considerable benefits, including weight loss and improved metabolic health. Despite potential side effects and the challenge of weight regain after discontinuation, these medications mark a critical step forward in addressing the global obesity crisis, emphasizing the importance of a comprehensive approach that combines medication with lifestyle adjustments for sustained health improvements.

### Side Effects of GLP-1 Agonist Medication Use

While GLP-1 agonists offer significant benefits through improved glycemic control and substantial weight loss, there are notable side effects that accompany medication use. If side effects are not understood by the patient, they can undermine the success of the medication. The most notable side effects include a notable calorie deficit, uncomfortable gastrointestinal symptoms, and potential loss of lean muscle mass.^34^

Glucagon-like peptide 1 receptor agonists promote weight loss by increasing a sense of satiety and reducing appetite, which decreases food intake and creates a calorie deficit that leads to weight loss.^17^ Patients taking these medications feel “full” for longer periods after eating due to slowed gastric emptying. Slow gastric function can create GI discomfort, including constipation, nausea, vomiting, and inhibit nutrient absorption.^17^^,^^22^^,^^35^ In addition to physical discomfort, the reduction in food intake and altered nutrient absorption can also lead to fewer minerals, vitamins, and essential nutrients being consumed.^36^

While a calorie deficit is necessary for weight loss, a prolonged calorie deficit can have adverse effects on energy expenditure,^34^ leading to decreased mood and energy level. Studies that examine prolonged calorie restriction report that participants show markedly less caloric expenditure from physical activity than their usual levels when in an energy deficit.^34^

A notable concern for people on GLP-1 agonist medications is loss of lean muscle mass.^37^ A substantial caloric deficit and insufficient protein intake, can lead to the body breaking down muscle tissue for energy. Loss of lean muscle mass leads to decreased muscular endurance, decreased strength, and increased risk for falls and rates of injury.^38^ If muscle mass could be maintained or even increased during weight loss, it may offset the reduction of metabolic rate that occurs with weight loss as well as improve glucose regulation.^37^

### GLP-1 Agonist Discontinuation

Recent data show a concerning trend in which 34% to 50% of patients stop using GLP-1 agonist medications within the first year, diminishing clinical benefits.^39^ There are several challenges that hinder access to these potentially life-altering treatments—primarily, high costs and medication shortages. This high rate of disruption in medication adherence underscores a need to further explore factors influencing medication adherence and identify supportive measures to enhance long-term outcomes.

Discontinuation of the medications affects appetite, metabolism and weight management. Weight regain is a primary concern when GLP-1 medications are discontinued.^29^ When the medication is discontinued the individual may experience increased hunger, return of food noise, and increased appetite making it difficult to control caloric intake which can result in weight gain. The individual’s metabolic rate will be decreased after use of GLP-1 agonists and subsequent weight loss or even maintaining weight loss will become more difficult.^15^^,^^16^ Further, after discontinuation some patients experience diminished glycemic control compared to when they were taking the medication. This happens because GLP-1 agonists help to improve insulin secretion and decrease glucagon secretion to improve overall glycemic control.^16^ When the medication is stopped patients experience deterioration of blood sugar regulation which may necessitate lifestyle interventions to mitigate the risk of returning to a chronic hyperglycemic state.^16^

## RELEVANCE TO PHYSICAL THERAPISTS 

As previously discussed, individuals with obesity and/or diabetes have an increased prevalence of musculoskeletal conditions.^4^^,^^5^ As such, it is imperative that physical therapists not only treat the musculoskeletal conditions, but also understand the effects of GLP-1 agonist medications and modify treatment plans to ensure safe and effective care. A holistic approach should be utilized, with care plans created in consideration of a patient’s current musculoskeletal condition, status of GLP-1 agonist medication use, current metabolic status and long-term health goals. This requires frequently assessing and adapting treatment plans to align with patients’ changing health status. Physical therapists should also recognize the importance of establishing sustainable physical activity routines to maintain health gains when the medications are discontinued.

## RECOMMENDATIONS FOR PHYSICAL THERAPISTS TREATING PATIENTS TAKING GLP-1 AGONIST MEDICATIONS

**Figure 4:**
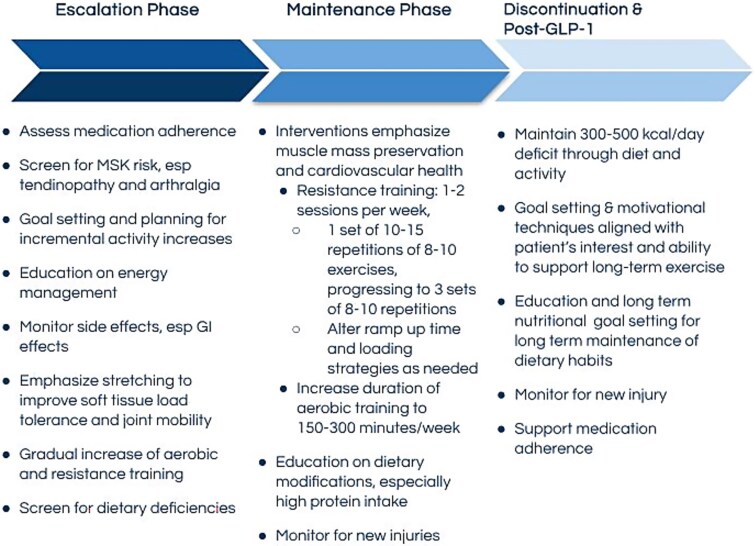
Physical Therapist Actions Throughout Typical Phases of GLP-1 Agonist Medication Use. During the escalation phase, which typically lasts 4 to 8 weeks, doses are increased gradually to minimize side effects and improve patient tolerance of the medication. When the optimal dose has been achieved, the patient will maintain medication use at a consistent level until they have achieved health goals or medication is discontinued. After the medication has been discontinued, the patient may experience a return of food noise, cravings, and increased appetite. However, to maintain health goals, the patient should maintain established exercise and diet changes to achieve a calorie deficit that allows them to maintain the health gains achieved through medication use.^40–42^ Abbreviations: GI = gastrointestinal; GLP-1 = glucagon-like peptide 1 receptor; MSK = musculoskeletal.

The effects of GLP-1 agonists require physical therapists to accommodate evolving health status and support patients in meeting their health goals. Physical therapists will play an integral role in the long-term success of patients as maintenance of the health benefits derived from GLP-1 agonist therapy necessitates lifestyle modifications like increasing activity levels and engaging in regular aerobic and resistance exercise. Physical therapist management strategies for patients taking GLP-1 agonist medications include detailed medical screening, early planning for muscle mass preservation, long-term health support, patient education and lifestyle modification, nutrition support, behavior change principles, and interdisciplinary collaboration. See Figure 4: Physical Therapist Actions Throughout Typical Phases of GLP-1 Agonist Medication Use for a summary of recommended physical therapist actions.

### Medical History Screening

It is imperative that physical therapists identify patients taking GLP-1 agonist medications at evaluation by including questions about this class of drugs as part of their medical history screening. Physical therapists must update medical history forms to include specific questions about GLP-1 agonist use, and confirm that the patient has obtained the referral from a licensed provider and filled the prescription at a reputable source. Because there are several ways to obtain these medications, including specialty digital health and wellness platforms, patients may obtain these medications without their primary care physician even being aware they are taking them. Therefore, physical therapists must be aware that GLP-1 agonist use may not be included in many patients’ electronic medical records.^43^ Given that up to 50% of patients discontinue GLP-1 agonist utilization within 12 months of beginning the medicaiton^39^ physical therapists should also evaluate medication adherence and persistence to support their health goals.

### Red Flag Screening

Red flag screening must also be updated. Traditionally, positive responses to questions about malaise, fatigue, bowel and bladder changes, and large changes in body weight would indicate a potential red flag and necessitate a referral to a primary care provider. However, these signs and symptoms are known side effects of GLP-1 agonist medications and a referral may not be necessary.^44^ When GLP-1 agonist medication use is known, providers can appropriately continue to provide supportive care in the presence of these symptoms provided there is no evidence or suspicion that the GLP-1 agonist medication is not the cause of the above signs and symptoms.

### Muscle Mass Preservation

There is strong evidence to support using resistance training and high protein diets as methods of preserving muscle mass after bariatric surgery, and current recommendations for patients taking GLP-1 agonists to employ similar strategies.^36^ A diet high in protein may preserve lean mass; therefore, protein intake of at least 60 to 75 g/day and up to 1.5 g/kg body weight/day is recommended.^36^^,^^37^ There may be benefits from increasing up to 2 or 3 times the recommended dietary allowance of 0.8 g/kg body weight/day for some individuals.^45^ Further, combining resistance exercise with increased protein intake can mitigate muscle loss even when in a calorie deficit.^33^ Evidence suggests 1 set of 10 to 15 repetitions for 8 to 10 exercises twice a week and progressing to 3 sets of 8 to 10 repetitions 3 times a week will build muscle mass with time and consistency.^32^^,^^46^ Additional studies are required to draw conclusions for best practices to preserve muscle mass for patients taking GLP-1 agonist medications, however consistent resistance training and high protein diets should be considered until further studies are completed.^33^

Lean muscle loss, combined with prolonged inflammation, rigid tendons, impaired muscle integrity, persistent atrophy, lipid accumulation, and joint degradation,^7^^,^^15^ will undermine musculoskeletal health, even as cardiometabolic health improves with GLP-1 agonist use. Because of these effects, patients taking GLP-1 agonist medications may need fewer sessions per week or longer ramp up periods with resistance training than never-obese counterparts. Loading strategies may need to be altered to accommodate for impaired joint, muscle or tendon function. It will be critical to monitor for new injuries, such as tendinopathies, as exercise habits are established.

### Support Long-Term Exercise Habits

GLP-1 agonists are intended to be used in conjunction with diet and exercise changes to deliver the best results.^47^ Education about the importance of adopting a sustainable, enjoyable, long term exercise routine for health maintenance is an integral component of a comprehensive physical therapy plan. As patients work through the escalation period and medication dose is gradually increased, they may experience very low energy. Therapists seeing patients in this phase may need to utilize motivational strategies to establish movement routines. Later, during the maintenance and discontinuation phase, patients may benefit from assistance with long-term goal setting to maintain exercise habits.

After GLP-1 agonist medications are discontinued, a caloric reduction of 300 to 500 kcal/day relative to baseline is required long term to maintain weight loss and health gains.^48^ As such, every care plan for a patient taking GLP-1 agonists should include both aerobic and resistance training. Current recommendations include 150 to 300 minutes of moderate intensity exercise and 2 to 3 sessions of resistance training per week.^49^ Meeting these requirements with an impaired musculoskeletal system is challenging, and physical therapists are uniquely trained to address these specific needs. Physical activity goals should be established with consideration to load management, muscle function, joint status, the individual’s overall health and activity abilities. Physical therapists can have a significant impact on the health outcomes of patients by educating patients on how to incorporate stretching, strengthening and cardiovascular exercise even in the presence of a compromised musculoskeletal system. When new musculoskeletal issues arise as activity levels increase, physical therapists have expertise to support patients continuing exercise in the presence of injury, thereby maintaining momentum toward health goals.

### Nutrition Screening

As most individuals in the United States do not meet recommendations for healthy dietary patterns,^50^ education regarding choosing nutrient dense foods and beverages is especially beneficial for patients taking GLP-1 agonist medications. Nutrition screening is within the scope of physical therapist practice^51^ and may provide significant benefit to this population when therapists identify poor dietary knowledge. Many self-report dietary screening tools are quick to administer, are widely available and provide guidance on easy strategies to improve diet, such as increasing fruit and vegetable intake. Additionally, nutritional screening may be beneficial to identify individuals at risk for deficiencies of iron, potassium, calcium, copper, magnesium, zinc as well as vitamins, such as vitamin A, C, D, E, K, and B.^36^ Physical therapists can provide reliable, evidence-based resources to patients, and support dietary behavior change.^52^

Research supports multiple strategies for consuming adequate protein to preserve or improve muscle mass, including consumption of plant-based proteins and supplementation with branched-chain amino acids or whey protein.^37^ The combination of resistance training and high protein intake are effective in maintaining lean mass during moderate caloric deficits.^45^ This should be a point of emphasis when educating our patients due to the functional and metabolic benefits of lean muscle mass. When necessary, utilizing multidisciplinary collaboration with registered dieticians to assist patients in improving eating habits to maximize the effectiveness of medication is appropriate.

### The Role of Behavior Change in GLP-1 Agonist Management

Physical therapists can play a pivotal role in the success of GLP-1 agonist medications by utilizing behavior change techniques to support increases in self-efficacy in exercise.^53^ Long term health changes require patients to adopt healthy lifestyles. Physical therapists should employ motivational interviewing techniques and goal setting strategies to assist patients in setting meaningful, achievable goals related to exercise and diet. By aligning physical therapist interventions to the patient’s interests and abilities, physical therapists can improve adherence and promote sustainable behavior change.^53^

Collaboration with other health professionals such as dieticians, primary care physicians and psychologists fosters a deeper understanding of the broad implications of GLP-1 agonist use. With education and support, physical therapists can help patients integrate new behaviors into their daily routines. Therapists should emphasize consistency with physical activity and self-management strategies, ensuring that behavior change principles are the foundation of all physical therapist interventions. Therapists may also consider referring patients to a health and well-being coach, in addition to other health care professionals, to support medication adherence and behavior change.^32^ This foundation will maximize the effectiveness of GLP-1 agonist medications and support long-term health and wellness.

## EMERGING TRENDS AND POTENTIAL PRACTICE IMPLICATIONS

Research on GLP-1 agonists suggests that these medications may be effective treatments for conditions beyond diabetes and obesity, suggesting a broad potential impact on health care. One such application is for chemical dependency, in which GLP-1 agonist is being investigated to decrease withdrawal effects and relapses. Other uses are being explored for Alzheimer, Parkinson disease, polycystic ovarian syndrome, infertility, sleep apnea, kidney disease and certain mental health disorders.^54^^,^^55^ While the treatment of many other diseases are being explored, there is conflicting evidence regarding the impact of GLP-1 agonists on areas that directly impact physical therapist practice such as OA and bone density.

Within orthopedic practice a number of important issues have yet to be fully explored. For example, GLP-1 agonist use has been associated with a higher rate of revisions for lumbar spinal fusions.^56^ While this study is small, it raises a number of questions for rehabilitation professionals regarding how GLP-1 agonist use may impact the prognosis for patients with musculoskeletal conditions. It brings about questions of safety for commonly used interventions such as manual therapy and joint mobilizations, and has the potential to change rehabilitation protocols for patients with surgical procedures like total joint replacements and open reduction internal fixation of fractures. There is conflicting evidence on the effect of GLP-1 agonists on bone health^57–61^ with some data suggesting weight loss with GLP-1 agonists reduces bone mineral density (BMD), but when combined with exercise BMD is maintained. Other data shows that GLP-1 agonists increase bone formation and prevent bone loss with weight loss emphasizing the fact that there is much to be learned regarding GLP-1 agonist use and musculoskeletal healing and rehabilitation. As it will likely be some time until we have definitive answers on how GLP-1 agonists influence musculoskeletal healing, clinicians must stay abreast of the developments and incorporate the best available information into their clinical reasoning process. Continued research is imperative to maximize and fully safely and effectively integrated into comprehensive orthopedic treatment strategies.

Prior research has demonstrated that weight loss through a variety of means (eg, bariatric surgery, diet and exercise, very low-calorie diets) results in 2% improvement in pain, function, and stiffness scores on the Western Ontario and McMaster Universities Osteoarthritis Index for every 1% weight loss.^62^ Similar results are obtained for individuals with obesity and knee OA who took GLP-1 agonists over 68 weeks in a recent study. On average they reduced their body weight by an average 13.7%, reported improved quality of life, a reduction in knee pain, a reduction in knee stiffness, and improvements in physical function.^63^ However, a large retrospective cohort study did not find differences in the progression to hip and knee OA in patients with obesity who were exposed to GLP-1 agonists compared with those who were not over a 5-year period. The same study also showed increased risk of progression to hip and knee OA in patients with diabetes, with or without obesity, who were exposed to GLP-1 agonists compared to patients with diabetes, with or without obesity, who were not exposed to GLP-1 agonists.^58^ Thus weight loss, whether mediated by GLP-1 agonists or other means, may be effective in improving symptoms in individuals with obesity and knee OA, but may not change the progression of knee and hip OA in the same population.

GLP-1 agonist use is likely to continue increasing, and therapists must stay abreast of the evolving uses and practice recommendations. Providers should be aware of the individual experience of their patients with these drugs and critically assess the unique health factors that impact outcomes when patients utilize this class of drugs. Therapists will need to be dynamic in their practice, enabling them to provide comprehensive care that integrates medication and lifestyle change for sustained health improvements.

## CONCLUSIONS

Physical therapists are vital in the health journey of those whom we serve, particularly those patients who are living with obesity, diabetes or both and using GLP-1 agonist medications. Given the prevalence of obesity and diabetes in the United States and the rapid adoption of GLP-1 agonists it is inevitable that physical therapists will treat patients using medications in this drug class. To provide the best care to patients and ensure overall health, there is an obligation to understand the impacts of these medications and recognize their profound effects on patients’ physical well-being. Physical therapists must meet this challenge by developing knowledge of the implications of these medications and leveraging their expertise in movement to realize the American Physical Therapy Association’s vision of “transforming society by optimizing movement to improve the human experience.”
